# EEG in the Emergency Department: When the Neurophysiological Test Can Be Avoided in Emergency Diagnostic Workups? The EMINENCE Study

**DOI:** 10.3390/neurolint18030054

**Published:** 2026-03-16

**Authors:** Maenia Scarpino, Antonello Grippo, Federica Barraco, Benedetta Piccardi, Laura Betti, Peiman Nazerian, Arianna Fabbri, Roberto Fratangelo, Cristina Mei, Andrea Nencioni

**Affiliations:** 1 Neurophysiopathology Unit, Careggi University Hospital, 50134 Florence, Italy; scarpinom@aou-careggi.toscana.it (M.S.); meic@aou-careggi.toscana.it (C.M.); 2 Emergency Department, Statale University Hospital, 20122 Milan, Italy; fbarraco@unimi.it; 3 Stroke Unit, Careggi University Hospital, 50134 Florence, Italy; piccardib@aou-careggi.toscana.it; 4 Emergency Department, Careggi University Hospital, 50134 Florence, Italy; bettil@aou-careggi.toscana.it (L.B.); nazerianp@aou-careggi.toscana.it (P.N.); arianna.fabbri@unifi.it (A.F.); nencionian@aou-careggi.toscana.it (A.N.); 5 UOC Neurologia, Ospedale San Giuseppe, 50053 Empoli, Italy; roberto.fratangelo@uslcentro.toscana.it

**Keywords:** emergency EEG (emEEG), emergency department, emergency diagnostic pathway

## Abstract

**Introduction**: This study was conducted to determine whether specific emergency physician (EP) diagnoses and/or neurological signs/symptoms upon admission to the Emergency Department (ED) were associated with normal/non-informative emergency electroencephalogram (emEEG). **Methods:** Data from consecutive patients admitted to the ED of our tertiary hospital over a two-year period (1 January 2023–31 December 2024) were analyzed retrospectively. We evaluated the correlation between normal/non-specific emEEGs and EP admission diagnoses and neurological signs/symptoms on admission. Epileptic discharges and sharp waves with triphasic morphology were considered specific patterns. **Results**: A total of 2008 patients underwent emEEG recording during the study period. EmEEGs were considered non-informative in 100% of global amnesia diagnoses, 100% of cases of mild head trauma, 100% of cases of migraine with aura, 98.3% of transient ischemic attacks (TIAs), 95.6% of transient losses of consciousness (TLCs) when seizure was not the primary suspected diagnosis, and in 92.7% of falls of unknown dynamics. Epileptic patterns were detected in 4% of patients presenting with TLC and in 2.4% of those with falls of unknown dynamics, with approximately half of these patients having a pre-existing diagnosis of epilepsy. Triphasic waves were detected in 4.9% patients with falls of unknown dynamics, in 1.7% with TIA, and in 0.4% with TLC. All of these patients had fever/sepsis or metabolic/electrolyte disorders. Overall, across all clinical scenarios, emEEGs were considered non-informative in 385 (19.1%) tested patients. **Conclusions:** emEEGs are almost non-informative in the diagnostic pathway for patients with global amnesia, mild head trauma, and migraine with aura, and in patients with TIA, TLC, or falls of unknown dynamics. EPs can safely consider avoiding emEEGs in the absence of previous epilepsy diagnosis, fever/sepsis, metabolic/electrolyte disturbances, or drug abuse.

## 1. Introduction

Electroencephalograms (EEGs) have been used to study brain function for almost 70 years [1]. Despite significant advancements in anatomical and functional neuroimaging, EEGs remain essential for evaluating specific pathological conditions, particularly those affecting consciousness, such as coma. Their ability to provide real-time insights into brain activity makes them an indispensable tool in clinical practice.

In addition to their long-standing use in outpatient epilepsy management, EEGs have recently become more widely used in emergency settings. This increase in use can be attributed to several factors: emergency EEGs (emEEGs) can be conveniently performed at the patient’s bedside, and they are rapid, non-invasive, and cost-effective. Most importantly, when neuroimaging results are negative, emEEGs can assist neurologists and emergency physicians in making a diagnosis. This benefit is particularly vital in acute situations where timely intervention can alter patient outcomes [2,3]. Furthermore, EEGs enable functional analysis of brain activity, complementing anatomical assessments conducted via neuroimaging techniques. While neuroimaging provides detailed images of brain structures, EEGs offer insight into the brain’s electrical activity, providing a more comprehensive understanding of a patient’s neurological status [2,3].

Although emEEGs can provide valuable information about brain function and aid in the diagnosis of several emergency conditions, including seizures and epileptic status (ES), both motor and non-convulsive [4,5,6,7,8], as well as confusional and altered mental states [9,10,11], there remains a significant gap in clinical practice regarding the absence of international guidelines governing their use.

This lack of guidance results in high demand for emEEGs in everyday clinical practice, often due to their performance characteristics and rapid availability or the individual experience of the treating physician instead of their actual diagnostic power.

Although a tertiary hospital can usually dedicate more than one neurophysiological technician and at least one EEG expert neurologist/neurophysiologist to recording and interpreting emEEGs during working hours, primary and secondary hospitals cannot meet such a high demand for emEEGs due to limited availability.

For this reason, in recent years, authors have focused on examining the contribution of emEEGs to the diagnostic and therapeutic workup of patients admitted to the emergency department (ED), attempting to identify the reasons why ED attending physicians order emEEGs and the clinical conditions or neurological signs and symptoms on admission for which they are most useful. This research seeks to provide evidence to inform recommendations for the use of emEEGs in daily clinical practice [1,2,3,11,12,13,14].

Conversely, identifying the clinical conditions usually associated with normal or non-informative EEGs performed as part of emergency workup can also contribute to establishing recommendations that standardize their use in the ED setting.

In a study by Scarpino et al. [2] conducted on a large sample of ED patients, normal emEEG results were observed in 32.1% of cases, particularly when the initial diagnosis was global amnesia or mild head trauma. Notably, none of these patients exhibited specific EEG abnormalities, such as epileptic discharges or triphasic sharp waves. This suggests that emEEG may not be necessary for patients with these initial diagnoses due to its non-specificity. Patients presenting to the ED following falls of unknown dynamics exhibited emEEG characteristics similar to those with global amnesia and mild head trauma. Only one patient with pre-existing epilepsy showed epileptic discharges, suggesting that emEEGs were indicated in patients with this initial diagnosis only if they had a positive epilepsy history. However, despite the large sample size of this previous study [2], the main limitation was represented by the small number of patients admitted to the ED with these specific initial diagnoses.

Therefore, this study was conducted to evaluate the correlation between normal or non-specific EEG abnormalities and the emergency physician’s primary diagnosis at the time of emEEG request, as well as neurological signs and symptoms on admission to the ED, in a greater number of patients. The specific objective was to identify clinical scenarios in which emEEGs may be safely omitted from the emergency diagnostic pathway due to their limited diagnostic yield.

## 2. Materials and Methods

The current study was an extension of the EMINENCE (**E**lectroencephalogra**M IN E**merge**NC**y d**E**partment) study [2].

EMINENCE was an observational, monocentric, retrospective study conducted at Careggi Teaching Hospital, a tertiary care center in Florence, Italy. The study included all patients over the age of 18 who were admitted to the ED and underwent emEEG recording between 1 January 2023 and 31 December 2024.

The main objective of the EMINENCE study [2] was to determine why ED physicians or consultant neurologists ordered emEEGs and their benefits, which were defined as changes in clinical diagnosis and the diagnostic–therapeutic management of patients, as well as subsequent discharge or hospitalization.

This report builds on the previous one-year EMINENCE study conducted at the same institution by adding an additional year of observational data. This provides more cases to analyze, enabling a more definitive evaluation of the benefit of emEEGs in various clinical scenarios.

Clinical data were extracted from medical records, including demographic and clinical variables such as gender, age, neurological signs and symptoms, and the indications for emEEG recording. The clinical context, including a history of epilepsy or other conditions predisposing the patient to ES or encephalopathy, was also recorded. Data on prior anti-seizure medications (ASMs) or other drugs/therapies that could predispose patients to neurological conditions assessed by emEEG, as well as records of neurological, neurosurgical, or resuscitator consultations and neuroimaging findings, were also collected. Additional variables, including electrolyte or metabolic imbalances, fever, septic state, and a history of hypertension, diabetes, dyslipidaemia, cardiac disorders, and thyroid disorders, were documented. Discharge or hospitalization was also recorded. We documented emEEGs recorded both during and outside normal hospital hours.

### 2.1. EEG Recording and Classification

EEG recording is available at our institution from 08:00 to 19:00 on weekdays and from 08:00 to 14:00 on Saturdays. Outside of these hours, EEG recording is available 24/7 for emergencies related to ES detection. Standard 30 min EEG recordings were performed using a 32-channels digital system Galileo-NT system (E.B. Neuro S.p.a., Florence, Italy) and an EEG prewired head-cap with 19 electrodes (Fp1, Fp2, F7, F8, F3, F4, C3, C4, T3, T4, P3, P4, T5, T6, O1, O2, Fz, Cz, and Pz) positioned according to the 10–20 international system. Recordings were acquired at a sampling rate of 128 Hz. During review, digital filters (low-pass filter = 30–70 Hz; time constant = 0.1–0.3 s; notch filter = 50 Hz) and sensitivity gain (2 to 10 V/mm, standard gain = 7 V/mm) were adjusted to meet interpretative needs [15,16,17,18].

EEG recordings were typically conducted without sedation. Interpretation was performed by expert neurologists or neurophysiologists as promptly as possible, with ED requests prioritized when necessary.

EEG classification followed the terminology established by the American Clinical Neurophysiology Society (ACNS) [19]. Descriptors included continuity, voltage, organization of the anterior–posterior gradient, reactivity, spontaneous variability of background activity, frequency, symmetry, epileptiform discharges, slow waves, periodic patterns, triphasic sharp waves, and the presence of sleep-related patterns. Seizure patterns, whether motor or electrical, as well as motor ES or non-convulsive ES (NCES), were also reported. Further details can be found in Hirsch et al. [19].

### 2.2. Outcome Assessment

We evaluated the correlation between normal or non-specific EEG abnormalities and the initial diagnosis and/or the neurological signs and symptoms present upon admission to the ED. We considered epileptic EEGs (including epileptic discharges, periodic patterns, and seizure/epileptic status) and EEGs showing triphasic sharp waves to demonstrate specific patterns because these EEG abnormalities enable a reliable diagnosis, even in the absence of other instrumental or biochemical data. This is in contrast to other EEG abnormalities, such as bilateral or focal slow waves or sharply contoured minor slow waves (theta), which, although they can contribute to a correct diagnosis, always require an association with other instrumental or biochemical information. The principal aim of this study was to identify patients for whom an emEEG recording might not be indicated in the emergency diagnostic–therapeutic pathway due to its non-specificity.

### 2.3. Statistical Analysis

Descriptive statistics were employed to define the study population, with categorical variables expressed as numbers and percentages and continuous variables as medians (interquartile range: 25th to 75th percentile).

Group comparisons were performed using chi-squared tests for categorical data and Kruskal–Wallis tests for continuous variables. Cramer’s V test was used to assess categorical correlations, with values between 0.30 and 0.49 indicating a moderate association and values above 0.50 signifying a strong correlation. All statistical analyses were conducted using Jamovi (version 2.6.2). Two-tailed tests were applied, and *p*-values < 0.05 were considered statistically significant.

### 2.4. Standard Protocol Approvals, Registrations, and Patient Consent

This study complied with ethical principles and good clinical practice guidelines. Due to logistical constraints, it was not feasible to obtain informed consent from all patients. Approval from the relevant ethics committee has been sought. The retrospective analysis of the patient database was approved by the local ethics committee (Ethics Committee Area Vasta Centro27241; Ethics Committee approval number: 27241_oss).

## 3. Results

A total of 211.844 patients were evaluated in the ED during the study period. In total, 13.795 patients were admitted for neurological symptoms, of whom 4610 had transient loss of consciousness (TLC).

A total of 2008 patients were included in the analysis, of whom 1018 (in the year 2023) were included in our previous EMINENCE study [2]. Of the 2008 patients analyzed, 211 (10.5%) underwent a second emEEG recording during their stay in the ED. The majority of the emEEGs (1984; 98.9%) were recorded during working hours.

In total, 1552 patients (77.4%) had an emEEG ordered directly by emergency physicians, while consultant neurologists ordered the neurophysiological test for the remaining 456 patients.

The median age of the cohort was 70 years [interquartile range (IQR) 29]. One thousand and four patients (50.0%) were male. Epilepsy had been previously diagnosed in 566 (28.1%) patents, of whom 353 had a structural etiology and 213 had an unknown etiology. Five hundred and ten patients were taking ASM.

One thousand and three hundred seventeen patients (65.5%) had no previous brain parenchymal damage. Of the remaining 691 patients, 238 (34.4%) had previously undergone neurosurgery. The most common causes of brain damage were previous ischemic stroke (185 patients; 26.7%), multi-infarct encephalopathy (137 patients; 19.8%), brain tumor (103 patients; 14.9%), previous traumatic brain injury (30 patients; 4.3%), and previous hemorrhagic stroke (25 patients; 3.6%). Three hundred sixty-four patients (18.1%) were admitted to the ED with metabolic or electrolyte disorders. Five hundred and seventy-six patients (28.7%) had a fever or sepsis, and 95 patients (4.7%) were admitted following drug abuse. Brain CT scans were performed on 1844 patients (91.8%), showing acute pathology in 297 patients (16.1%). The most common type of brain damage was cerebral ischemia (50 patients, 16.8%). The demographic characteristics of the patients are shown in Table 1.

On admission, neurological examinations revealed involuntary movements in 715 patients (35.6%), TLC in 687 patients (34.2%), cognitive/behavioral impairment in 672 patients (33.4%), speech disorder in 512 patients (25.4%), focal motor impairment in 282 patients (14.0%) and an altered level of consciousness in 267 patients (13.2%). Meanwhile, 1296 patients (64.6%) showed a resolution of the symptoms that led to their admission to the ED. Of all the patients, 628 (31.3%) were subsequently admitted to the hospital, 49 (2.4%) refused admission, and the remaining 1331 (66.3%) were discharged home after their ED visit. emEEG recording and interpretation were performed by the neurologist/neurophysiologist with a median time of 90 min (IQR 75 min) after the ED attending physician’s request.

The most common clinical indications for an emEEG were seizures (520; 25.8%) or suspected seizures (263; 13.0%), followed by TLC (227; 11.3%), altered mental status (158; 7.8%), ischemic or hemorrhagic stroke (124; 6.1%), also in cases of their differential diagnosis with seizure or post-critical state (111; 5.5%), and transient ischemic attack (TIA) (118; 5.8%). The remaining clinical indications, in order of frequency, were ES (61, 3.0%), global amnesia (55, 2.7%), mild head trauma (45, 2.1%), falls of unknown dynamics (41, 2.0%), and migraine with aura (15, 0.7%).

In 250 cases (12.4%), there was no defined clinical indication, and patients underwent emEEG recording depending on the presence of neurological symptoms or signs such as cognitive or behavioral impairment (215 cases, 86%), speech disorders (80 cases, 32%), and headache (41 cases, 16.4%).

Table 2 shows a comprehensive list of emEEG characteristics according to the ED admission diagnosis, with a normal emEEG pattern found in 653 patients (32.5%), predominantly in subjects admitted for migraine with aura (12, 80%), global amnesia (42, 76.4%), TLC when seizures were not highly suspected (144, 63.4%), mild head trauma (25, 55.6%), TIA (62, 52.5%), and falls of unknown dynamics (17, 41.5%).

Table 3 provides a comprehensive list of EEG characteristics according to the neurological signs and symptoms observed upon admission to the ED, showing that all the neurological signs/symptoms, except for hallucinations, were associated with the presence of a specific emEEG pattern (usually epileptic abnormalities), which accounted for more than 5% of the total.

Table 4 shows the distribution of specific and non-specific emEEGs and the respective final diagnoses according to the ED admission diagnoses usually associated with a non-specific emEEG. For example, patients with an initial diagnosis of falls of unknown dynamic were discharged with different final diagnoses—18 patients with confirmed falls of unknown etiology, 3 with encephalopathy, and 17 with other diagnoses—when the emEEG was normal or non-specific. When the emEEG showed epileptic discharges (in one patient), the final diagnosis was seizure. In the two patients with triphasic sharp waves, the final diagnosis was septicemia.

**Figure 1:**
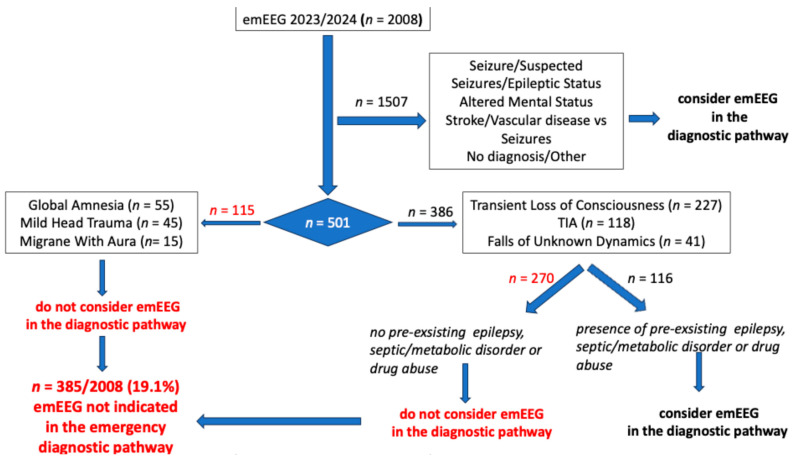
A practical suggestion for identifying patients for whom an emEEG would not be necessary as part of the emergency diagnostic pathway. Red type indicates the patients not requiring an emEEG.

Figure 1 illustrates a practical suggestion for identifying patients for whom an emEEG would not be mandatory in the emergency diagnostic pathway. An emEEG recording is not mandatory for patients admitted for migraine with aura (*n* = 15), global amnesia (*n* = 55), or mild head trauma (*n* = 45) because none of these patients’ emEEGs showed a specific pattern. Of the remaining admission diagnoses that are usually, but not always, associated with a non-informative emEEG (TLC without a high suspicion of seizures, TIA, and falls of unknown dynamics), 66 of 227 (29.0%) patients admitted for TLC, 22 of 118 (18.6%) patients admitted for TIA, and 28 of 41 (68.2%) patients admitted for falls of unknow dynamics had pre-existing epilepsy or metabolic/septic derangements that could potentially cause encephalopathic disorders. Thus, of the 386 patients with these three admission diagnoses, an emEEG could be indicated for 116 (30%). The probability of identifying specific EEG abnormalities in these patients is at least twice as high as in the overall patients admitted to the ED for TLC, TIA, and falls of unknown dynamics. Thus, among the 2008 patients admitted, emEEG recording was not necessary in the emergency diagnostic pathway of 385 patients (19.1%) due to its lack of informativeness. These 385 patients included all 115 patients admitted for global amnesia, mild head trauma, and migraine with aura and 270 patients admitted for TLC, falls of unknown dynamics, and TIA.

## 4. Discussion

This large retrospective study demonstrates that several ED scenarios in which the emEEGs requested are consistently associated with non-informative findings. In particular, emEEGs did not provide relevant information in the emergency diagnostic pathway of patients presenting with global amnesia, mild head trauma, or migraine with aura. In patients presenting with TLC, falls of unknown dynamics, or TIA, emEEGs were rarely informative unless specific risk factors were present, such as a history of epilepsy or evidence of metabolic disorder, sepsis, or drug abuse. These findings suggest that emEEGs should be used selectively rather than requested routinely in these scenarios.

The term “emergency” refers to a life-threatening pathological condition that requires immediate treatment. Neurological disorders that cause an altered state of consciousness are usually included among emergent conditions, and many of them (e.g., NCES, metabolic or septic encephalopathy and encephalitis) can be detected on an emEEG. The usefulness of emEEGs in emergency clinical practice is well-established. Specifically, emEEGs are beneficial during the first 24–48 h after the onset of neurological symptoms. They are useful not only for the clinical management of ES, NCES, and ASM changes but also for patients with loss of consciousness, acute altered mental status, mental confusion, and ischemic lesions. This remains the case even when neuroimaging results are normal [1,7,11,13,14,20,21,22]. However, there are still no universally accepted recommendations for the use of emEEGs except for the management of NCES and motor ES [1,4,5,6,7,8,12]. The lack of guidelines on the use of emEEGs is mainly due to scattered data in the literature and heterogeneous sample selection, recruitment, and settings, which make it difficult to compare reported results. Previous studies on the subject were limited by small sample sizes [1,7,11,13,23,24] and the heterogeneity of their settings, with most studies [1,7,23,24] including intensive care units (ICUs). Consequently, several emEEGs were ordered to detect brain death or predict the neurological outcome for patients with severe brain injuries [15,18]. Even when the ICU was not involved, inpatient wards were included, resulting in EEG requests from neurology and neurosurgery wards, where the attending physicians have more expertise in selecting neurophysiological tests [11].

To provide recommendations for the use of emEEGs in the emergency diagnostic pathway, it is important to limit evaluations to the ED because it follows a specific daily workflow that differs from that in inpatient wards and is even more different than the ICU setting.

The absence of universally accepted emEEG guidelines has resulted in widespread emEEG use to meet the ED’s specific needs. This leads to over-prescription and misuse, reducing the emEEG’s diagnostic power and specificity.

Therefore, in ED clinical practice, it is important to identify the admission diagnoses and/or neurological signs/symptoms that are most likely to be associated with informative/specific EEGs performed in emergencies, as well as those that are usually associated with normal or non-specific EEG abnormalities. This would optimize the use of available instrumental tests, costs, and healthcare professional resources without negatively impacting the emergency diagnostic pathway for patients.

To the best of our knowledge, this is the first study in the literature to focus on this topic. In a previous paper, Scarpino et al. [2] reported ED admission diagnoses usually associated with normal or non-specific emEEGs. These diagnoses were particularly prevalent in cases of global amnesia, mild head trauma, and the majority of cases involving falls of unknown dynamics, with the exception of one patient with pre-existing epilepsy. This suggests that an emEEG recording may not be necessary in subjects with these initial clinical scenarios due to its non-specificity.

However, despite the fact that the study by Scarpino et al. [2] was conducted on a large sample of patients, it was limited by the fact that only a small number of patients were admitted to the ED with these specific initial diagnoses.

For this reason, we conducted a study with a larger sample size than our previous work [2] to confirm and strengthen our findings, with the aim of identifying patients for whom emEEG recording may not be indicated in the emergency diagnostic-therapeutic pathway due to its non-specificity.

Our data analysis revealed that the most common admission diagnoses associated with a non-specific emEEG were global amnesia (100%), mild head trauma (100%), migraine with aura (100%), transient ischemic attack (TIA) (98.3%), TLC when seizures are not highly suspected (95.6%), and falls of unknown dynamics (92.7%). Notably, all of these admission diagnoses were associated with either the absence or presence of specific EEG patterns (both epileptic and triphasic), accounting for less than 5% of cases. No patients admitted for global amnesia, mild head trauma, or migraine with aura exhibited specific emEEG patterns. Epileptic patterns were detected in 4.0% of patients admitted for TLC and in 2.4% of those admitted for falls of unknown dynamics; half of these patients had pre-existing epilepsy. Triphasic morphology sharp waves were detected in 4.9% of patients with falls of unknown dynamics, 1.7% of patients admitted for TIA, and 0.4% of patients admitted for TLC. All of these patients presented with fever, sepsis, metabolic/electrolyte disorders, or drug abuse.

Thus, according to our results, and as shown in Figure 1, of the 2008 patients included in this analysis, 501 (25%) showed an absence or, alternatively, the presence of specific EEG patterns in fewer than 5% of cases. More specifically, an emEEG may not be mandatory in the emergency diagnostic pathway for patients admitted to the ED with a diagnosis of global amnesia, mild head trauma, or migraine with aura, as it was not informative in these cases (for a total of 115 patients, 23%). Of the remaining 386 patients (77%), who corresponded to subjects admitted for TLC, falls of unknown etiology and TIA, an emEEG could only provide information in 116 cases (30%). This concerned patients with pre-existing epilepsy or septic/metabolic disorders upon admission to the ED that might determine signs of brain involvement such as septic or metabolic encephalopathy.

Thus, according to our results, of the 2008 patients, 385 (19.1%) could not have an emEEG recording as part of the emergency pathway because it would not be informative.

Our results demonstrate the importance for ED physicians of making the most accurate admission diagnosis possible based on admission symptoms and anamnestic data, even though this can often be difficult in an ED setting, where initial information is frequently incomplete. However, accurate diagnosis upon admission could significantly impact the benefit patients derive from certain diagnostic tests. This is particularly true in primary and secondary care hospitals, where the availability of diagnostic tests and staff is limited. An illustrative example is the difference between an admission diagnosis of TLC in the absence of suspected seizures and an admission diagnosis of suspected seizures when not witnessed. According to our results, only 4% of patients with an admission diagnosis of TLC showed an epileptic pattern on emEEG, whereas the percentage of specific emEEGs with epileptic discharges increased to 48.7% when the admission diagnosis was suspected seizures.

Considering an admission diagnosis of TLC as an example, the reduced usefulness of the emEEG, which usually but not always showed non-specific patterns, was also evident from its lower contribution to the final ED diagnosis of these patients (Table 4). In contrast to most other admission diagnoses, where the emEEG result had a significant impact on the final ED diagnosis, our data analysis revealed that 6 of the 215 patients who were admitted for TLC and had non-specific EEGs were discharged with a suspected seizure diagnosis, while 5 were discharged with an infectious or metabolic/electrolyte disorder diagnosis. At the same time, of the 9 patients with epileptic emEEGs, 7 were discharged with a TIA diagnosis, and only 2 were discharged with a seizure diagnosis. Similar results were observed for patients admitted for TIA: 75 out of 116 subjects with non-specific emEEGs were discharged with heterogenous final diagnoses, including seizures and infectious or metabolic/electrolyte disorders. These results suggest that the ED physicians relied more on admission signs, symptoms, and anamnestic data than on emEEG results for the final diagnosis of these patients.

According to our data, when considered individually, no neurological admission signs or symptoms were associated with non-informative EEGs except in patients who experienced hallucinations. This provides further evidence of the importance for ED physicians of making an accurate admission diagnosis by considering patients’ neurological and other clinical signs and symptoms and their medical history. When a specific diagnosis could not be made, cognitive/behavioral impairment, speech disorders, and headache were the most frequent neurological symptoms on admission for which an emEEG was requested. Of these emEEGs, 11.5% were specific for epileptic patterns. Therefore, according to our results, performing an emEEG recording in an emergency setting could contribute to achieving a final diagnosis in patients for whom a reliable diagnosis could not be made upon admission.

It is also important to note that patients admitted for seizures or suspected seizures and are subsequently discharged from the ED with an epileptic etiology may exhibit an EEG characterized by the absence of epileptic discharges, with abnormalities that are more akin to those of the non-specific EEGs associated with other diagnostic suspicions or even normal emEEGs. This finding is consistent with the previous literature, which indicated that the diagnostic value of an emEEG for witnessed or suspected seizures is just over 50% [5,25]. The substantial difference in the diagnostic pathway of these patients in whom emEEG recording is necessary compared to those admitted for loss of consciousness without high suspicion of seizure is represented by the percentage of specific epileptic emEEGs, which was 41.7% and 48.7% for seizures and suspected seizures, respectively, and less than 5% for patients admitted for TLC. These results highlight the importance of making an accurate admission diagnosis. This enables ED physicians to not only identify patients who could benefit most from an emEEG but also ensure the correct interpretation of emEEG results, even when they are non-specific or normal.

Finally, our data demonstrated that when an accurate admission diagnosis is made, ED physicians feel confident enough to omit EEG recording from the emergency pathway when specific neurological conditions are suspected, since the results of the neurophysiological test would not typically provide information that could affect patient management in the ED. In these cases, an EEG recording could be included in a fast-track outpatient program to complete the diagnostic workup. For ED patients, this would reduce the cost and length of hospital stays associated with the EEG recording, time spent waiting for the neurophysiological report, and neurological consultation. While these costs and timeframes may not be extremely relevant in a third-level hospital, where resources and qualified personnel are sufficient, they could have a significant impact in primary and secondary hospitals, where resources are limited.

Although this study is the first to focus on ED admission diagnoses or neurological signs/symptoms usually associated with a normal/non-specific EEG, thus contributing evidence to support recommendations for daily use of emEEGs in clinical practice, particularly in the ED, it has some limitations. Primarily, its retrospective design meant that incomplete clinical and instrumental information was collected from medical records, and it was not possible to determine how the ED physicians used the emEEG’s diagnostic information or its exact contribution to the final diagnosis. To achieve the most objective results possible, we examined the distribution of specific and non-specific emEEGs and the final diagnosis according to the ED admission diagnoses, particularly those usually associated with a non-specific emEEG (Table 4). We observed that in most cases, emEEG patterns were consistent with the final diagnosis. This was particularly true for admission diagnoses that were always associated with a non-informative emEEG (global amnesia, mild head trauma, and migraine with aura), whereas some patients admitted for the remaining admission diagnoses (mainly TLC and TIA) were discharged with more heterogenous final diagnoses, including seizures and infectious or metabolic/electrolyte disturbances, despite the presence of a non-specific emEEG pattern (Table 4). This suggests that ED physicians relied more on admission signs, symptoms, and anamnestic data than on the emEEG result when making the final diagnosis for these patients. Another limitation was the monocentric setting of a tertiary hospital, which treats many complex and severe cases that may not be representative of those in smaller hospitals. Furthermore, in this tertiary hospital, in which at least two or three neurophysiological technicians and one EEG expert neurologist/neurophysiologist were dedicated exclusively to recording and interpreting emEEGs during working hours, limited the generalizability of the work, particularly with regard to primary and secondary hospitals where emEEG availability is limited.

This study, which included a large sample of subjects and limited the evaluation to emEEGs performed in the ED, attempted to identify patients for whom an emEEG recording may not be indicated in the emergency diagnostic–therapeutic pathway due to its non-specificity, meaning that it could not provide additional crucial information that could significantly modify the management of patients in an emergency setting. By employing an appropriately sized sample, our study has the potential to be the first to comprehensively evaluate non-informative emEEGs in emergency settings. Previous research in this area has been limited by small patient cohorts and heterogeneous environments, making it difficult to generalize findings to broader populations. By addressing these gaps, our study aims to clarify the utility of emEEGs in acute medical scenarios. In particular, it provides evidence to inform recommendations for daily clinical practice, especially in specific settings such as the ED.

## 5. Conclusions

For EEG recording to be effectively integrated into emergency care, it is essential to establish international guidelines that standardize its use. These guidelines should provide a clear framework for when and how EEGs should be utilized in emergency contexts, ensuring that their application is efficient and beneficial. This includes cases in which this neurophysiological test is not expected to provide information, meaning it is unnecessary in an emergency setting.

It would be of great importance to develop these recommendations using well-established methodological tools, not only for the management of ES but also to identify cases in which emEEGs are non-informative. This would be particularly beneficial for primary and secondary hospitals where emEEG availability is limited, resources are scarce, and highly qualified personnel are needed.

## Figures and Tables

**Table 1:** Patients’ demographic characteristics (*n* = 2008).

Characteristics	
Age year, median (IQR)	70 (29)
Male gender, *n* (%)	1004 (50.0%)
Previous Epileptic Seizures	566 (28.1%)
*Unknown aetiology*	*213 (10.6%)*
*Structural aetiology*	*353 (17.6%)*
Antiseizure medications	510 (25.4%)
Under-dosed antiseizure medications	122 (6.1%)
Fever	262 (13.1%)
Sepsis	314 (15.6%)
Metabolic disturbance	129 (6.4%)
Electrolyte disturbance	235 (11.7%)
Drug abuse	95 (4.7%)
Previous Neurological history	691 (34.4%)
*Stroke*	*159 (7.8%)*
*Neurosurgery*	*238 (11.9%)*
Cardiac disorders	462 (23.0%)
Diabetes	327 (16.3%)
Dyslipidemia	540 (26.9%)
Thyroid disease	218 (10.9%)
Brain CT	1844 (91.8%)
*Recent CT Lesions*	*297 (14.7%)*
Home discharge	1331 (66.3%)
Hospitalization	628 (31.3%)
Hospitalization Refused	49 (2.4%)

**Table 2:** Emergency EEG characteristics and abnormalities according to diagnosis on admission.

	Normal	Epileptiform	Triphasic Sharp Waves	Frequency Reduced	No Reactivity	Asymmetry	Theta	Delta
**Cramer’s V** **ED admission diagnosis**	**0.280**	**0.421**	0.158	0.170	0.255	0.173	0.203	0.179
**Seizure (*n* = 520)**	96 (18.5)	217 (41.7)	7 (1.3)	197 (37.9)	218 (41.9)	111 (21.3)	246 (47.3)	270 (51.9)
**Suspected Seizures (*n* = 263)**	84 (31.9)	64 (48.7)	7 (2.6)	99 (37.6)	74 (28.1)	50 (19.0)	105 (39.9)	17 (6.4)
**Epileptic Status (*n* = 61)**	3 (4.9%)	41 (67.2)	0 (0.0)	34 (55.7)	42 (68.8)	30 (49.2)	32 (52.5)	50 (81.9)
**Altered Mental Status (*n* = 158)**	20 (18.4)	24 (27.3)	21 (13.2)	109 (68.9)	123 (77.8)	28 (17.07)	35 (22.1)	80 (50.6)
**TLC (*n* = 227)**	144 (63.4)	9 (4.0)	1 (0.4)	41 (18.0)	19 (8.3)	8 (3.6)	32 (14.1)	28 (12.3)
**TIA (*n* = 118)**	62 (52.5)	0 (0.0)	2 (1.7)	26 (22.0)	9 (7.9)	4 (3.4)	27 (22.9)	22 (18.6)
**Stroke (*n* = 124)**	34 (27.4)	11 (8.8)	8 (6.4)	57 (45.9)	48 (35.8)	24 (19.3)	35 (28.2)	56 (45.1)
**Vascular disease vs. seizures (*n* = 111)**	24 (21.6)	25 (22.5)	0 (0.0)	49 (44.1)	36 (32.4)	30 (27.0.)	56 (50.5)	53 (47.7)
**Falls Of Unknown Dynamics (*n* = 41)**	17 (41.5)	1 (2.4)	2 (4.9)	19 (46.3)	12 (29.3)	2 (4.9)	7 (17.1)	12 (29.2)
**Global Amnesia (*n* = 55)**	42 (76.4)	0 (0.0)	0 (0.0)	0 (0.0)	2 (3.6)	0 (0.0)	8 (14.5)	6 (10.9)
**Mild head Trauma (*n* = 45)**	25 (55.6)	0 (0.0)	0 (0.0)	10 (22.2)	8 (17.8)	4 (8.9)	11 (24.4)	8 (17.7)
**Migraine with Aura (*n* = 15)**	12 (80.0)	0 (0.0)	0 (0.0)	0 (0.0)	0 (0.0)	0 (0.0)	2 (13.3)	1 (6.7)
**No Diagnosis/Other (*n* = 250)**	90 (36.0)	28 (11.5)	7 (2.9)	99 (39.6)	69 (27.6)	40 (16.4)	68 (27.2)	82 (32.8)
**Total (*n* = 2008)**	653 (32.5)	420 (20.9)	55 (2.7)	740 (36.8)	660 (32.8)	331 (16.4)	664 (33.1)	414 (20.6)

TLC: Transient Loss of Consciousness; TIA: Transient Ischemic Attack; ED: Emergency Department.

**Table 3:** Emergency EEG characteristics and abnormalities according to neurologic signs/symptoms on patient’s admission.

	Normal	Epileptiform	Triphasic Sharp Waves	Frequency Reduced	No Reactivity	Asymmetry	Theta	Delta
Neurological Signs/Symptoms on Admission	*n* (%)	*n* (%)	*n* (%)	*n* (%)	*n* (%)	*n* (%)	*n* (%)	*n* (%)
**TLC (*n* = 687)**	265 (38.6)	142 (20.7)	6 (0.9)	232 (33.7)	30 (4.4)	85 (12.4)	243 (35.4)	232 (33.7)
**Language Disorder (*n* = 512)**	126 (24.6)	92 (18.0)	13 (2.5)	238 (46.4)	195 (38.1)	114 (22.2)	192 (37.5)	235 (45.8)
**Confusion (*n* = 477)**	134 (28.1)	81 (17.0)	22 (4.6)	230 (48.2)	198 (41.5)	74 (15.5)	153 (32.1)	197 (41.2)
**Motor Manifestation (*n* = 715)**	159 (22.2)	264 (36.9)	15 (2.1)	212 (29.7)	234 (32.7)	97 (13.6)	302 (42.2)	365 (51.0)
**Altered Mental Status (*n* = 267)**	24 (9.0)	72 (27.0)	29 (10.8)	206 (77.1)	209 (78.2)	60 (22.5)	88 (33.0)	159 (59.5)
**Absence (*n* = 124)**	34 (27.4)	33 (26.6.)	5 (4.0)	50 (40.4)	34 (33.0)	23 (18.2)	51 (41.1)	53 (41.7)
**Behavioral Impairment (*n* = 195)**	46 (23.5)	41 (21.0)	12 (6.1)	116 (59.4)	100 (51.2.)	35 (17.9)	73 (37.4.)	94 (48.2)
**Sensory Symptoms (*n* = 140)**	72 (51.4)	25 (17.9)	2 (1.4)	17 (19.2)	26 (18.6)	20 (14.3)	38 (27.1)	38 (27.1)
**Hypostenia (*n* = 282)**	70 (24.8)	62 (22.0)	5 (1.7)	104 (36.8)	119 (42.1)	77 (27.3)	113 (40.1)	134 (47.5)
**Headache (*n* = 148)**	77 (52.0)	23 (15.5)	1 (0.6)	21 (14.1)	33 (22.3)	20 (13.5)	46 (31.1)	37 (25.0)
**Asthenia (*n* = 53)**	24 (45.3)	4 (7.5)	2 (3.7)	13 (24.5)	18 (33.9)	8 (15.0)	17 (32.1)	17 (32.1)
**Amnesia (*n* = 197)**	107 (54.3)	23 (11.7)	1 (0.5)	28 (14.2)	30 (15.2)	11 (5.5)	57 (28.9)	37 (18.7)
**Hallucinations (*n* = 22)**	7 (31.8)	1 (4.5)	1 (4.5)	9 (40.9)	7 (31.8)	7 (31.8)	5 (22.7)	8 (36.3)
**Catatonic State (*n* = 18)**	4 (22.2)	3 (16.7)	1 (5.5)	9 (50.0)	8 (44.4)	2 (11.1)	9 (50.0)	6 (33.3)
**Visual Disturbance (*n* = 66)**	18 (27.1)	16 (24.6)	0 (0.0)	18 (27.2)	29 (44.6)	18 (27.2)	23 (35.4)	28 (42.4)
**Morsus (*n* = 139)**	40 (28.8)	46 (33.1)	1 (0.7)	36 (25.8)	41 (29.5)	13 (9.3)	59 (42.4)	59 (42.4)
**Postural Instability (*n* = 39)**	17 (43.6)	4 (10.3)	0 (0.0)	17 (43.6)	13 (33.3)	4 (10.3)	10 (25.6)	8 (20.6)

TLC: Transient Loss of Consciousness.

**Table 4:** Distribution of specific and non-specific emEEGs and the respective final diagnoses according to the ED admission diagnoses usually associated with a non-specific emEEG.

	Discharge Diagnosis			
Diagnosis at Patient Admission (*n*)	Confirmed Diagnosis	Seizures/ Suspected Seizure	Encephalopatic Disease	Others Diagnosis
**TLC (227)**				
Normal/non-specific emEEG (*n* = 217)	178	6	5	26
Epileptic emEEG (*n* = 9)	7	2	0	0
Triphasic emEEG (*n* = 1)	0	0	1	0
**TIA (118)**				
Normal/non-specific emEEG (*n* = 116)	41	6	2	67
Epileptic emEEG (*n* = 0)	0	0	0	0
Triphasic emEEG (*n* = 2)	0	0	2	0
**Falls Of Unknow Dynamics (41)**				
Normal/non-specific emEEG (*n* = 38)	18	0	3	17
Epileptic emEEG (*n* = 1)	0	1	0	0
Triphasic emEEG (*n* = 2)	0	0	2	0
**Global Amnesia (55)**				
Normal/non-specific emEEG (*n* = 55)	50	0	0	5
Epileptic emEEG (*n* = 0)	0	0	0	0
Triphasic emEEG (*n* = 0)	0	0	0	0
**Head Trauma (45)**				
Normal/non-specific emEEG (*n* = 45)	37	0	0	8
Epileptic emEEG (*n* = 0)	0	0	0	0
Triphasic emEEG (*n* = 0)	0	0	0	0
**Migraine with aura (15)**				
Normal/non-specific emEEG (*n* = 15)	14	0	0	1
Epileptic emEEG (*n* = 0)	0	0	0	0
Triphasic emEEG (*n* = 0)	0	0	0	0

TLC: Transient Loss of Consciousness; ED: Emergency Department; TIA: Transient Ischemic Attack.

## Data Availability

The raw data supporting the conclusions of this article will be made available by the authors upon request.

## Abbreviations

The following abbreviations are used in this manuscript:

**ACNS**	American Clinical Neurophysiology Society
**ASM**	Antiseizure Medication
**CT**	Computed Tomography
**ED**	Emergency Department
**EEG**	Electroencephalogram
**emEEG**	Emergency EEG
**ES**	Epileptic Status
**ICU**	Intensive Care Unit
**IQR**	Interquartile Range
**NCSE**	Non-convulsive Epileptic Status
**TIA**	Transient Ischemic Attack
**TLC**	Transient Loss of Consciousness
